# Sialic acid-binding Ig-like lectin-7 interacts with HIV-1 gp120 and facilitates infection of CD4^pos^ T cells and macrophages

**DOI:** 10.1186/1742-4690-10-154

**Published:** 2013-12-13

**Authors:** Stefania Varchetta, Paolo Lusso, Kelly Hudspeth, Joanna Mikulak, Dalila Mele, Stefania Paolucci, Raffaello Cimbro, Mauro Malnati, Agostino Riva, Renato Maserati, Mario U Mondelli, Domenico Mavilio

**Affiliations:** 1 Research Laboratories, Department of Infectious Diseases, Fondazione IRCCS, Policlinico San Matteo, Pavia, Italy; 2 Laboratory of Immunoregulation, National Institute of Allergy and Infectious Diseases, National Institutes of Health, Bethesda, MD, USA; 3 Unit of Clinical and Experimental Immunology, Humanitas Clinical and Research Center, Rozzano, Milan, Italy; 4 S. S. Virologia Molecolare, S. C. Virologia e Microbiologia, Fondazione IRCCS Policlinico San Matteo, Pavia, Italy; 5 Unit of Human Virology Division of Immunology, Transplantation and Infectious Diseases, San Raffaele Scientific Institute, Milan, Italy; 6 Dipartimento di Scienze Cliniche (DISC) L. Sacco Hospital-Infectious diseases and Immunopathology Section, University of Milan, Milan, Italy; 7 HIV Outpatient clinic, Department of Infectious Diseases, Fondazione IRCCS Policlinico San Matteo, Pavia, Italy; 8 Department of Internal Medicine, University of Pavia, Pavia, Italy; 9 Department of Medical Biotechnologies and Translational Medicine, University of Milan, Milan, Italy

**Keywords:** HIV-1 infection, Siglec-7, CD4+ T cells, Macrophages, AIDS patients

## Abstract

**Background:**

Sialic acid-binding Ig-like lectin-7 (Siglec-7) expression is strongly reduced on natural killer (NK) cells from HIV-1 infected viremic patients. To investigate the mechanism(s) underlying this phenomenon, we hypothesized that Siglec-7 could contribute to the infection of CD4^pos^ target cells following its interaction with HIV-1 envelope (Env) glycoprotein 120 (gp120).

**Results:**

The ability of Siglec-7 to bind gp120 Env in a sialic acid-dependent manner facilitates the infection of both T cells and monocyte-derived macrophages (MDMs). Indeed, pre-incubation of HIV-1 with soluble Siglec-7 (sSiglec-7) increases the infection rate of CD4^pos^ T cells, which do not constitutively express Siglec-7. Conversely, selective blockade of Siglec-7 markedly reduces the degree of HIV-1 infection in Siglec-7^pos^ MDMs. Finally, the sSiglec-7 amount is increased in the serum of AIDS patients with high levels of HIV-1 viremia and inversely correlates with CD4^pos^ T cell counts.

**Conclusions:**

Our results show that Siglec-7 binds HIV-1 and contributes to enhance the susceptibility to infection of CD4^pos^ T cells and MDMs. This phenomenon plays a role in HIV-1 pathogenesis and in disease progression, as suggested by the inverse correlation between high serum level of sSiglec-7 and the low CD4^pos^ T cell count observed in AIDS patients in the presence of chronic viral replication.

## Background

The human immunodeficiency virus type 1 (HIV-1) envelope (Env) glycoprotein 120 (gp120) is extensively covered by carbohydrates that play an active role in the viral life cycle. Indeed, besides forming a protecting shield from antibody (Ab) recognition, this coat of N-linked glycans affects the folding of viral glycoproteins and virus infectivity [1-4]. gp120 N-linked glycans include 11 high-mannose or hybrid-type glycans and 13 complex-type glycans (containing terminal sialic acids) that are highly conserved in different isolates and clades [3,5]. Sialic acids are a family of sugars with a 9-carbon backbone present at the ends of glycan chains in all cell types [6,7]. They are recognized by many sugar-binding proteins, including sialic acid-binding lectins (Siglecs), a family of 16 I-type lectins showing a specific affinity for different sialic acids. In particular, Siglec-7 preferentially binds α(2,8)-linked disialic acid and α(2,6)-linked sialic acid [8]. Siglec-7 is constitutively expressed on human natural killer (NK) cells, monocytes and a small subset of CD8^pos^ T cells [9-13]. In regard to interactions with pathogens, Siglec-7 has been shown to bind *Campylobacter jejuni* and *Pseudomonas aeruginosa* expressing or displaying sialic acids on their surface, respectively [14-16].

Many human lectin-type receptors including galectins [17,18], defensins [19-21] and others [22-27] have been shown to bind HIV-1 Env by recognizing glycans expressed on gp120. However, these molecules may affect HIV-1 infection in an opposite way as some, such as mannose-binding lectin (MBL) [22] and langerin [23], inhibit HIV-1 infection, while others, such as galectin-1, DC-SIGN [24], mannose receptor [25], syndecan-3 [26] and DCIR [27], increase the susceptibility to HIV-1 infection.

Recently, it has been shown that different Siglecs, such as Siglec-1 and Siglec-7, recognize HIV-1 and enhance infection of monocytes [28], macrophages [29] and dendritic cells (DCs) [30]. Indeed, sialic acids present on Env gp120 can be recognized by Siglecs either expressed on these immune cells or released in soluble forms, thus facilitating viral entry into target cells. Although Siglec-7 has been shown of being able to bind Env gp120 from different HIV-1 strains with lower affinity if compared to Siglec-1 [29], very little is know about the role of Siglec-7 in participating to the HIV-1 infections of CD4^pos^ target cells. We have previously shown that the expression of Siglec-7 is significantly reduced on the surface of NK cell from HIV-1 infected viremic patients and that the successful suppression of viral replication by antiretroviral therapy (ART) restores Siglec-7 expression on these cells [31].

The present study demonstrates that, following its binding with HIV-1 Env gp120, Siglec-7 contributes to viral entry and infection of both CD4^pos^ T cells and monocyte-derived macrophages (MDMs). Indeed, our results demonstrate that the treatment with soluble Siglec-7-Fc fusion protein increases the susceptibility to HIV-1 infection in Siglec-7^neg^/CD4^pos^ T cells, while blockade of Siglec-7 with a specific Ab reduces the degree of infection in Siglec-7^pos^ MDMs. Finally, the study shows that in the sera of viremic AIDS patients there are increased serum levels of soluble Siglec-7 (sSiglec-7) that inversely correlates with CD4^pos^ T cell counts, thus suggesting a direct role of this glycan-binding protein in the modulation of HIV-1 infection and disease progression.

## Results

### Soluble Siglec-7 binds HIV-1 envelope gp120 recombinant protein from different HIV-1 strains

**Figure 1:**
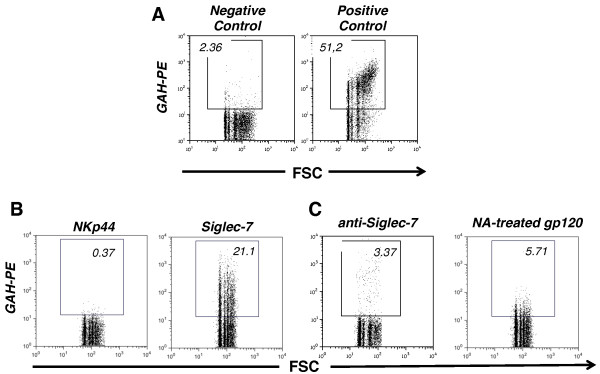
**Siglec-7 binds recombinant env gp120. A**, Dot plot flow cytometry graphs showing representative examples of negative and positive controls for the binding experiments reported in the 1B and 1C panels. The negative control graph (left) illustrates the absence of interactions between env gp120 from HIV-1 IIIB strain covalently conjugated to carboxyl microparticles and phycoerythrin (PE)-labeled goat anti-human (GAH). The positive control shows the binding of env gp120 from HIV-1 IIIB strain covalently conjugated to carboxyl microparticles to PE-labeled anti-env gp120 mAb (clone 2G12). **B**, Dot plot flow cytometry graphs showing a representative example of the binding of NKp44 (left) or Siglec-7 (right) Fc fusion proteins to env gp120 from HIV-1 IIIB strain covalently conjugated to carboxyl microparticles. The binding was detected trough a PE-labeled GAH Fc Ab conjugated to the fusion proteins. **C**, Dot plot flow cytometry graphs showing a representative example of the binding of Siglec-7 Fc fusion protein to env gp120 from HIV-1 IIIB strain covalently conjugated to carboxyl microparticles in the presence of a pool of anti Siglec-7-specifc antibodies (left) and of gp120 neuraminidase treatment (right).

**Figure 2:**
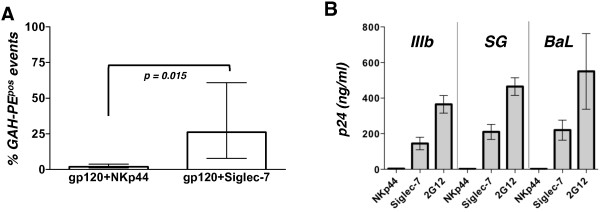
**Siglec-7 interacts with recombinant env gp120 from different HIV-1 strains. A**, Histogram bar graph showing cumulative results representative of 7 different experiments (mean ± SD), whose representative example is showed in Figure 1B. **B**, Histogram bar graph showing the amount of p24 core antigen detected by ELISA as indicative of the bindings of NKp44 and Siglec-7 Fc fusion proteins to different HIV-1 viral strains. Data are representative of 2 independent experiments (mean ± SD). The binding of R5 or X4 HIV-1 isolates to beads coated with the anti-gp120 mAb (clone 2G12) was used as positive internal control.

On the basis of our previous report [31] showing a significant reduction in Siglec-7 expression on NK cells from HIV-1 viremic patients, we proceeded to confirm whether Siglec-7 could directly interact with HIV-1 Env gp120 [29]. To this end, recombinant gp120 from HIV-1 IIIB virus (produced in mammalian CHO cells) was conjugated to carboxyl microparticles. We then evaluated the ability of this complex to bind Siglec-7-Fc protein by flow cytometry. As internal negative control, we used NKp44 Fc chimera. We observed that the only Siglec-7 chimera, and not the NKp44 one, has the ability to bind gp120 (Figures 1A-B and 2A). The pre-incubation of Siglec-7 Fc fusion protein with a pool of 2 different anti-Siglec-7 monoclonal Abs (mAbs) (QA79 and Z176 clones) resulted in an inhibition of the binding (Figure 1C, left panel), thus demonstrating the specificity of the Siglec-7-HIV-1 interaction. Moreover, this binding appeared to be sialic acid-dependent, since pre-treatment of gp120 with neuraminidase (NA) strongly inhibits the interaction between Siglec-7 and the HIV-1 envelope protein (Figure 1C, right panel).

The ability of Siglec-7 to directly bind HIV-1 was then assessed by using both R5 (BaL, SG) and X4 tropic (IIIB) isolates in a virion-binding assay. Goat anti-human Fc magnetic beads coated with Siglec-7-Fc or NKp44-Fc proteins were incubated with the HIV-1 infectious viral stocks, and the binding was evaluated by measuring the amount of captured p24 HIV-1 antigen by ELISA. In line with the experiments shown in the panels A-B-C of Figure 1, we found detectable amounts of p24 only in the presence of Siglec-7-Fc fusion protein coated beads and not after incubation with beads coated with NKp44-Fc chimera, regardless of the HIV-1 strains used. The binding of R5 or X4 HIV-1 isolates to beads coated with the anti-gp120 mAb 2G12 was used as positive internal control (Figure 2B).

### Siglec-7 fusion protein enhances HIV-1 infection of CD4^pos^ T cells

**Figure 3:**
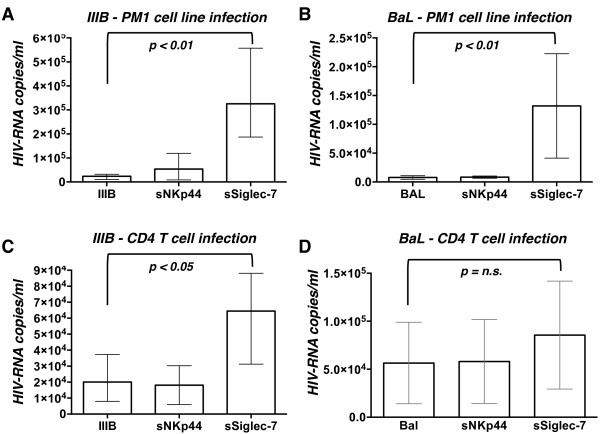
**Pre-incubation of soluble Siglec-7 protein with HIV-1 virus increases the infection of CD4**^**pos**^**T cells.** Histogram bar graph showing the copies/ml of HIV-RNA transcripts in the cell supernatant of PM1 cell line **(A-B)** and CD4^pos^ primary T cells **(C-D)** infected with IIIB **(A-C)** or BaL **(B-D)** viral strains pre-incubated or not with either NKp44 or Siglec-7 fusion proteins. Data are representative of 3 independent experiments (mean ± SD).

Since we observed that Siglec-7 is able to bind gp120, we sought to determine if this interaction could contribute to HIV-1 infection at the cellular level. We first choose as target PM1, a CD4^pos^ T cell line known to be susceptible to infection by different HIV-1 isolates, including primary CCR5- and CXCR4-tropic tropic strains [32,33]. We incubated for 1 hour the R5 (BaL) and X4 (IIIB) HIV-1 viral stocks with Siglec-7- or NKp44- Fc chimeras and subsequently we infected PM1 cells. The degree of infection was evaluated after 3 days of culture. PM1 cells infected with either IIIB or BaL strains pre-incubated with Siglec-7 chimera showed a significant increase in HIV-RNA copies compared to their counterparts infected with untreated viral isolates (Figure 3A-B). No effects were observed when PM1 cells were infected with HIV-1 strains pre-incubated with NKp44 Fc fusion protein, thus confirming again the specificity of Siglec-7 in enhancing the susceptibility to HIV-1 infection.

We then investigated whether the Siglec-7 chimera was able to amplify the HIV-1 productive infection also in primary CD4^pos^ T cells, the main target of HIV infection [34,35]. To this end, we followed the same protocol used for PM1 cells. Again, our results showed a statistically significant increase of HIV-1 viral load in CD4^pos^ T cells infected with HIV-1 IIIB pre-incubated with Siglec-7-Fc chimera compared to their counterpart infected with untreated HIV-1 strains, while pre-incubation of viral isolates with NKp44 fusion protein did not enhance the degree of infection of CD4^pos^ T cells (Figure 3C). Although, we observed a higher HIV-1 viral load also in CD4^pos^ T cells infected with HIV-1 BaL pre-incubated with Siglec-7-Fc chimera, this increase did not reach a statistical significance (Figure 3D).

### Siglec-7 fusion protein increases HIV-1 viral entry in primary CD4^pos^ T cells

**Figure 4:**
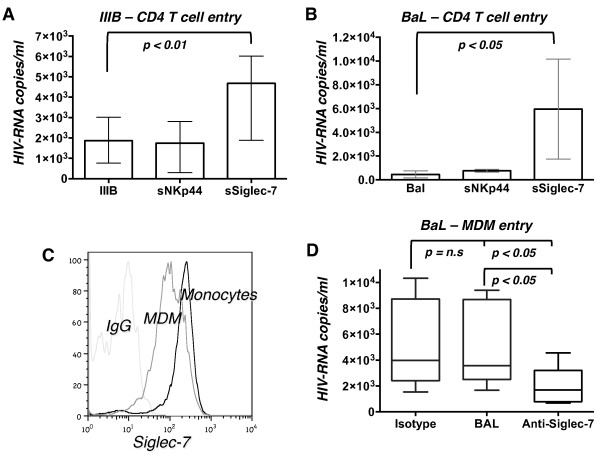
**Soluble Siglec-7 facilitates HIV-1 entry in CD4**^**pos**^**T cells and MDMs. A-B**, Histogram bar graph showing the copies/ml of HIV-RNA transcripts in the CD4^pos^ primary T cell lysates infected with IIIB **(A)** or BaL **(B)** viral strains either pre-incubated or not with either NKp44 or Siglec-7 fusion proteins. **C**, Histogram flow cytometric graph from a representative example showing the surface expression of Siglec-7 on monocytes (black line) and on MDMs (grey line) compared to isotype control (light gray line). **D**, Histogram bar graph showing the copies/ml of HIV-RNA transcripts in the MDM cell lysates infected with BaL viral strain either in the absence or in the presence of a blocking polyclonal antibody anti-Siglec-7. The related isotype control is included in the graph. Data are representative of 4 independent experiments (mean ± SD).

To understand if the binding of Siglec-7 with HIV-1 gp120 protein effectively contributes to viral entry process into target cells, we performed a 4-hour entry assay in CD4^pos^ T cells infected with either BaL or IIIB viral strains pre-incubated with Siglec-7-Fc or NKp44-Fc fusion proteins. The number of HIV-RNA copies detected in cell lysates was significantly increased in CD4^pos^ T cells infected with IIIB and BaL strains pre-incubated with Siglec-7-Fc protein but not in cells pre-treated with NKp44-Fc chimera compared with control infections (Figure 4A-B).

T cells are generally considered not to express substantial amounts of Fcγ receptors (FcγRs) although there are a few reports showing a positive expression of CD64 (FcγRI), CD32 (FcγRII) and CD16 (FcγRIII) on some CD4^pos^ cell line or unique primary T cell subsets [36,37]. Since our experimental approach relies on recombinant Siglec-7-Fc fusion protein as a surrogate of sSiglec-7, we proceeded to demonstrate that the enhanced HIV entry/infection in CD4^pos^ T cell targets by Siglec-7-Fc is not associated with the expression of FcγRs. To this end, we measured the surface levels of CD64, CD32 and CD16 on PM1 cell lines and primary CD4^pos^ T cells by flow cytometry and we did not detect any significant or bimodal expression of FcγRs on the aforementioned populations compared to positive controls (Additional files 1, 2 and 3).

### Anti-Siglec-7 blocking Ab inhibits HIV-1 viral entry in MDMs

MDMs are susceptible to HIV-1 infection as they express both CD4 and the co-receptors CCR5 and CXCR4 [38,39]. However, unlike primary CD4^pos^ T cells and similar to circulating monocytes [13], MDMs constitutively express high levels of Siglec-7 (Figure 4C), thus representing a useful cellular model to study the Siglec-7/HIV-1 interaction. Since we found that sSiglec-7 promotes HIV-1 entry into CD4^pos^ T cells, we asked whether blockade of this lectin-type receptor on the surface of MDMs could reduce the degree of HIV-1 infection. To validate this hypothesis, MDMs were cultured with HIV-1 BaL either with or without a previous incubation with anti-Siglec-7 blocking Ab. The amount of HIV-RNA transcripts was evaluated in MDM lysates 4 hours after infection to assess viral internalization. The results showed that the masking of Siglec-7 induced a statistically significant decrease of HIV-RNA copies compared to the infection performed in the absence of anti-Siglec-7 blocking Ab (Figure 4D).

### Serum levels of sSiglec-7 correlate with viral load in HIV-I infected patients naïve for antiretroviral therapy (ART)

**Figure 5:**
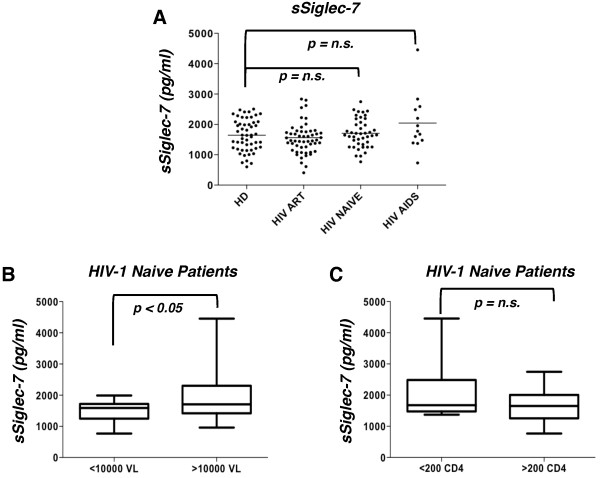
**Serum level of soluble Siglec-7 is associated to viral load and CD4**^**pos**^**T cell counts. A**, Dot plot graph showing the level of serum Siglec-7 levels in healthy donors (HD), naïve, ART treated and AIDS patients (mean). **B**, A significant increase in serum Siglec-7 levels was observed in naïve patients with viral load >10,000 copies/ml. **C**, sSiglec-7 was decreased in patients with CD4^pos^ T cell cell count lower than 200/mm^3^.

Given the ability of the soluble form of this lectin-type receptor in enhancing the susceptibility to HIV-1 infection in target cells, we then investigated whether or not sSiglec-7 plays a role in the natural course of HIV-1 infection by quantifying its levels in the serum of 42 HIV seropositive naïve patients, 13 AIDS patients, 54 aviremic patients treated with antiretroviral therapy (ART) compared to 52 healthy donors (Table 1). We did not detect any significant difference in serum levels of sSiglec-7 between healthy donors and the different cohorts of HIV-1 infected patients. However, AIDS patients displayed a trend toward increase of serum Siglec-7 compared to healthy donors (Figure 5A). Moreover, a significant increase of sSiglec-7 was observed in the sera of naïve patients with viral load above 10,000 copies/ml compared with naïve patients with viral load below this level (Figure 5B). Although not statistically significant, a similar trend toward increase of serum levels of sSiglec-7 was observed in naïve patients with a CD4^pos^ T cell count lower than 200/mm^3^ compared with those with CD4^pos^ T cell counts higher than 200/mm^3^ (Figure 5C).

**Table 1:** Demographic and clinical features of HIV-1 infected patients

	**NAIVE¶**	ART*	**AIDS#**	**TOTAL**
	**(n = 42)**	**(n = 54)**	**(n = 13)**	**(n = 109)**
Gender				
*Female*	8	14	3	25
*Male*	34	40	10	84
Age (years)				
*Median*	46	52	51	50
*Range*	22-53	38-66	47-75	22-75
Plasma HIV-1 RNA (copies/ml)				
*Median*	16816	<50	46630	1620
*Range*	74-309691	0-412137	22025-402137	0-402137
CD4^pos^ T cell count (cells/mm^3^)				
*Median*	380	491	111.5	386.5
*Range*	99-1042	98-1328	3-197	3-1328

^¶^Naïve patients for antiretroviral therapy (ART) without a clinical history of opportunistic infections or malignancies associated to HIV-1 infection.

^*^ART included at least one protease inhibitor, one non-nucleoside reverse-transcriptase inhibitor and /or two nucleoside reverse-transcriptase inhibitors.

^#^Chronically HIV-1 infected patients either naïve for ART or therapeutic regimen had been discontinued at least 24 months earlier. Clinical history included opportunistic infections or malignancies associated to HIV-1 infection.

**Figure 6:**
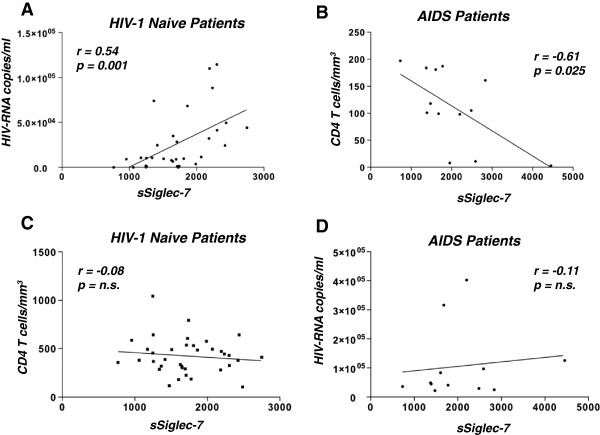
**Correlations between sSiglec-7, CD4 cell counts and HIV-1 viral loads. A**, Statistical analyses showing the correlation between the levels of sSiglec-7 in the serum of naïve patients and HIV-1viremia. **B**, Statistical analyses showing the correlation between the levels of sSiglec-7 in the serum of AIDS patients and CD4^pos^ T cell count. **C**, Statistical analyses showing the correlation between the levels of sSiglec-7 in the serum of naïve patients and the CD4+ T cell count. **D**, Statistical analyses showing the correlation between the levels of sSiglec-7 in the serum of AIDS patients and HIV-1 viremia.

Interestingly, the levels of sSiglec-7 directly correlated with the HIV-1 viral load but not with the CD4^pos^ T cell count in untreated patients, while AIDS patients showed an inverse correlation between the amount of sSiglec-7 and the CD4^pos^ T cell count. No correlation was found between sSiglec-7 and HIV-RNA in AIDS patients (Figure 6).

## Discussion

In this study, we show that the direct interaction between Siglec-7 and HIV-1 Env gp120 contributes to HIV-1 entry and infection of CD4^pos^ cell targets, including T cells and macrophages. These findings are consistent with recent studies reporting that the interactions between sialic acids present on the viral envelope and Siglec receptors facilitate HIV-1 infection in several cellular targets [28-30].

Siglec molecules have been recognized as ligands of different sialic-acid bearing pathogens [13-15,40-44], although it is still debated whether resting leukocytes with masked Siglecs are able to directly engage sialic acids on pathogens or if cell activation is required for unmasking these lectin-type receptors. Originally, the interactions between Siglec receptors and pathogens were reported to dampen host immune responses and to set appropriate activation thresholds for regulating cell proliferation and secretion of inflammatory mediators. Subsequently, it became evident that this biological phenomenon represents an advantage for pathogens that evolved to express sialic acids on their surface to evade host innate immune responses [45,46]. However, recent evidence showed that sialic acid-expressing pathogens could also promote infection and mediate immune responses following their binding to Siglecs [13,47,48].

In line with a previous report [29], we demonstrate that Siglec-7 is able to directly interact with HIV-1 Env gp120 although we found a certain degree of variability among different HIV-1 strains. This phenomenon may have several explanations, including deletions or mutations of critical residues involved in Siglec-7 binding or, most likely, heterogeneity in HIV-1 glycosylation patterns [1-4,13]. Evidence in favor of the latter hypothesis comes from our results showing that Siglec-7 interaction with HIV-1 is indeed sialic acid-dependent. In this regard, it has also been reported that sialic acid depletion from human CD4^pos^ target cells enhances HIV-1 infection [49]. This suggest that removing cis-interactions between sialic acids and Siglecs on the same cell leads to exposure of Siglecs that, in turn, become accessible to sialic acids exposed on gp120. Moreover, oligomeric modeling of the gp120 trimer shows that complex glycans are exposed on the outer rim, potentially accessible to Siglec recognition [50]. Furthermore, it has recently been shown that different Siglecs, and Siglec-1 in particular, are able to recognize both R5 and X4 HIV-1 gp120 through interaction with sialic acids, thus contributing to HIV-1 infection of monocytes, macrophages and DCs [28-30].

The present study also provides evidence that Siglec-7 binds HIV-1 and enhances viral entry into CD4^pos^ T cells when the soluble form of this lectin-type molecule is added to the culture. Indeed, our data clearly showed that Siglec-7-Fc fusion protein is able to enhance IIIB and BaL infections both in a CD4^pos^ T cell line (PM1) and in primary CD4^pos^ T cells, even though the levels of HIV-RNA were higher for IIIB compared to BaL. This latter phenomenon is likely due to the well-known preferential tropism of the IIIB HIV-1 strain for T cells [51]. Further investigations focusing on the structures, biochemical properties and affinity of these interactions are needed to confirm this working hypothesis. Moreover, we also demonstrate that constitutive expression of Siglec-7 on MDMs markedly contributes to their HIV-1 infection, since specific Siglec-7 blockade induced a reduction of HIV-RNA in MDMs in a 4-hour entry assay. The degree of surface levels of Siglec-7 on monocytes and macrophages is still being debated, as there is one study showing that expression of this molecule on monocytes and MDMs is very low [29], while several other reports demonstrated that monocytes express high levels of Siglec-7 [9,11,12,46,52]. In our experiments, the constitutive expression of Siglec-7 on monocytes paralleled the ones observed on NK cells [13], and remained very high on MDMs even after 7 days of culture with GM-CSF although at lower levels if compared to freshly isolated monocytes.

Given the ability of Siglec-7 fusion protein to enhance in vitro HIV-1 infection of CD4^pos^ T cells, we then determined whether different levels of sSiglec-7 released during the course of HIV-1 infection are associated with different clinical outcomes. We did not find any significant differences in the serum levels of sSiglec-7 between healthy controls and several different cohorts of HIV-1 infected patients. However, AIDS patients showed a trend toward increase of Siglec-7 in their sera compared to healthy donors and, in particular, we found a direct and statistically significant correlation between HIV-RNA and serum levels of Siglec-7 in naïve HIV-1 infected patients, suggesting that a higher viral burden could induce the shedding of Siglec-7 in serum. In this regard, it is conceivable to hypothesize that a possible source of sSiglec-7 are NK cells, since we demonstrated that the exposure of these cells to high chronic HIV-1 viremia induce the expansion of pathological NK cell subsets showing an aberrant receptor repertoire and an unusual negative expression of Siglec-7 [53,54]. The fact that Siglec-7 fusion protein enhances the *in vitro* susceptibility of CD4^pos^ T cells to be infected by HIV-1 is also consistent with the persistent high levels of HIV-1 viremia, with the high amount of sSiglec-7 and with low CD4^pos^ T cell counts found in AIDS patients. Interestingly, a CD56^neg^/Siglec-7^neg^ population associated with high levels of HIV-1 viremia has been described as a dysfunctional NK cell subset that is significantly expanded in the late stages of HIV-1 infection [31]. In agreement with this, sSiglec-7 was found to be significantly increased in the sera of patients with viral load over 10,000 copies/ml, suggesting a possible role for this lectin-type receptor as a biological marker involved in the disease progression. We are currently testing this hypothesis in a larger patient cohort. Finally, an inverse correlation between the serum levels of Siglec-7 and CD4^pos^ T cell counts was observed in AIDS patients, which were also the cohort of HIV-1 infected subjects with the highest serum levels of sSiglec-7. These findings lead us to hypothesize that the presence of remarkable amounts of sSiglec-7, together with a low CD4^pos^ T cell count, high levels of HIV-1 viremia and expansion of Siglec-7^neg^ NK cells, characterize the advanced stages of HIV infection.

For our experimental approaches testing the ability sSiglec-7 to enhance the infection of CD4^pos^ PM1 cell line and primary T cells, we used a dose of fusion protein (10 μg/ml) much higher compared to amount of sSiglec-7 found in the sera of both healthy donors and HIV-1 infected patients (floating within a ng/ml range). We have chosen to use a higher sSiglec-7 concentration in our in vitro approach to maximize ligand-receptor binding. However, it must be emphasized that identical concentrations of an irrelevant protein such as soluble NKp44 was unable to promote infection using the same experimental conditions. Moreover, even though the serum concentrations of sSiglec-7 in HIV-1 infected patients or healthy donors are remarkably lower compared to those used in our in vitro approach, it remains to be determined the amounts of this soluble protein during acute phases of infection when HIV-1 viral load is in the ranges of several hundred-thousand copies [31]. Finally, we need to assess whether higher levels of sSiglec-7 are also observed at gut or cervix mucosal levels, two of the main gates of viral entrance [55,56]. Indeed, macrophages as well as NK and T cells play an active role within these tissue sites in context of innate and adaptive immune responses and establishment of viral reservoirs in the CD4^pos^ targets [57-59].

## Conclusions

**Figure 7:**
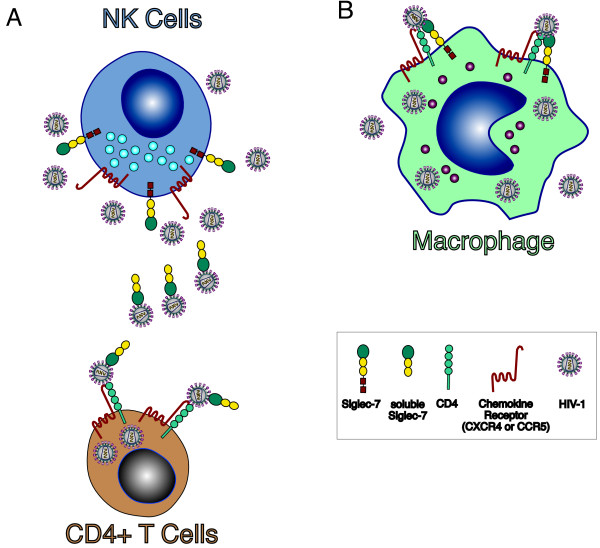
**Siglec-7 and susceptibility to HIV-1 infection. A**, Chronic and high levels of HIV-1 replication in AIDS patients lead to the decreased surface expression of Siglec-7 on NK cells [31] and to higher levels of soluble Siglec-7 detected in the plasma of HIV-1 infected patients with elevated viral burden (Figures 5 and 6). HIV-1 is able to bind Siglec-7 (Figure 1) that, in its soluble form facilitates the infection of Siglec-7^neg^/CD4^pos^ T cells (Figures 3 and 4). **B**, In contrast, high levels of viral replication do not affect the constitutive expression on monocytes and macrophage of Siglec-7 [14-31], which in turns, significantly contribute to HIV-1 infection of these cells. These experimental evidence suggest that, similar to other members of Siglec family [28-30], Siglec-7 greatly enhances either in its soluble form or as membrane receptor the susceptibility of CD4^pos^ cell targets expressing chemokine receptors to be infected by HIV-1, while spares from infection NK cells that, although positive for CCR5 or CXCR4, do not express CD4 [54].

Our study shows that HIV-1 infection of target cells is modulated by Siglec-7, which both enhances viral entry in Siglec-7^neg^/CD4^pos^ T cells in its soluble form and participates to the infection of Siglec-7^pos^ MDMs (Figure 7). These results, together with the positive correlations between sSiglec-7 and viral load in the sera of HIV-1 infected naïve patients and with our previous data showing a pathologic expansion of highly dysfunctional CD56^neg^/Siglec-7^neg^ cells in AIDS patients [31], prompt us to hypothesize that this lectin-type molecule plays an important role in HIV-1 pathogenesis by being associated with an higher susceptibility of CD4^pos^ target cells to be infected and with disease progression. The disclosure of these novel insights might lead to the use of Siglec-7 as potential biomarker during the course of HIV-1 infection and to therapeutically target this lectin-type receptor in order to inhibit viral replication/spreading thus limiting the depletion of CD4^pos^ T cells and the establishment of viral reservoirs.

## Methods

### Patients

Peripheral blood mononuclear cells (PBMCs) were obtained from buffy coat in accordance with the clinical protocol approved by the Institutional Review Board (IRB) of San Matteo Hospital, Pavia, Italy. The sera of HIV-1 infected patients (Table 1) were obtained in accordance with the clinical protocol approved by the IRB of Sacco Hospital, Milan, Italy. Donors from both protocols signed consent forms that were approved by the above-mentioned IRBs in accordance with Italian laws and with Declaration of Helsinki.

### Reagents

Recombinant human Siglec-7 and NKp44 fusion proteins and blocking anti-Siglec-7 polyclonal Ab were purchased from R&D system Inc. Commercially available ELISA kits were used for the detection of HIV-1 gp120 (Perkin Elmer) and sSiglec-7 (DuoSet, R&D system). Recombinant gp120s and anti-gp120 HIV-1 monoclonal Ab (mAb) 2G12 were obtained from the National Institutes of Health AIDS Research and Reference Reagent Program. Anti-Siglec-7 monoclonal Abs (mAbs) QA79 and Z176 were purchased from Instrumentation Laboratories.

### Protein-beads conjugation

Gp120 from HIV-1 IIIB strain was covalently conjugated to carboxyl microparticles using the PolyLink Protein coupling kit (Bangs Laboratories Inc.) following the manufacturer instructions. Siglec-7-Fc or NKp44-Fc fusion proteins were incubated with BioMag particles coated with goat anti-human IgG Fc Abs (Qiagen) in order to bind the beads.

### Siglec-7 binding to gp120

260 ng of gp120-conjugated beads (130 μg/ml) were incubated with 1 μg of Siglec-7-Fc or NKp44-Fc protein for 30’ at RT. After three washing, a phycoerythrin (PE) polyclonal goat anti-human-Fc was added for 30’ at 4°C. Samples were then washed and analyzed with a Facscalibur flow cytometer (BD Biosciences). Data were analyzed using FlowJo software (TreeStar).

Sialidase treatment was performed incubating 0.16 units/ml of *Vibrio cholerae* neuraminidase (Sigma) in DMEM medium with gp120 coated beads for 2 hours at 37°C. Flicking of the tubes was done every 20’ to get a uniform treatment of sialidase. Beads were washed three times with DMEM 10% FCS.

### Virus binding assay

10 μg of Siglec-7-Fc or NKp44-Fc proteins conjugated to magnetic beads were incubated with viral stocks of different HIV-1 strains (IIIB, SG, BaL; 5 × 10^5^ copies/ml). After three washing steps with complete, culture medium samples were solubilized in lysis buffer (250 μl of 0.5% NP40 alternative, Calbiochem-Merck), bead debris were removed by magnetic adhesion and the concentration of p24 protein released in the buffer was measured by ELISA. The binding of R5 or X4 HIV-1 isolates to beads coated with the anti-gp120 mAb 2G12 was used a positive internal control.

### Cells

PM1 cells were cultured in RPMI 1640 medium (Sigma) supplemented with 10% fetal calf serum (HyClone South Logan), 2 mM L-glutamine (Sigma) and antibiotics (100 U/ml penicillin, 0.1 μg/ml streptomycin, 0.25 μg/ml amphotericin B, Sigma). CD4+ T cells and CD14+ monocytes were isolated from PBMCs by negative magnetic selection (EasySep, Stemcell) from PBMCs obtained by healthy donors by standard density gradient centrifugation (Lympho Separation Medium, MP Biomedicals). The purity of both populations was always higher than 95%. Monocytes were cultured at 5 × 10^5^ cells/ml in 48-well plates in RPMI 1640 supplemented with 10% fetal bovine serum (HyClone South Logan), 10% human serum AB (EuroClone S.p.A.), 2 mM L-glutamine (Sigma), antibiotics (100 U/ml penicillin, 0.1 mg/ml streptomycin, 0.25 mg/ml amphotericin B, Sigma) and 10 ng/ml GM-CSF for 7 days with medium replacement every 3 days. We analyzed the surface expression of Siglec-7, CD14 and CD33 (BD Pharmigen) on MDMs after 8 days of culture, in the presence of 20% human serum to avoid non-specific binding to Fc-receptors.

### Flow cytometry

For multicolour (up to 8 colours) (FACS Fortessa, BD Pharmigen) flow cytometric analyses, PBMCs were stained with Brilliant Violet 421-labeled CD56, fluorescein isothiocyanate (FITC)-labeled CD3, Pacific Blue-labeled CD14 (Biolegend), allophycocyanin(APC)-labeled CD4 (R&D system), PerCP-labeled CD8, APC/C7-labeled 19, PC7-labeled CD16, phycoerythrin(PE)-labeled CD32 and CD64 (BD Pharmigen) monoclonal antibodies (mAbs). Viable cells were detected through the aqua live/dead staining, according to manufacturer instructions (Invitrogen). Within the lymphocyte gate, CD4^pos^ T cells were defined as CD3^pos^, CD19^neg^, CD56^neg^, CD8^neg^ cells, while monocytes were defined as CD14^pos^ cells within the related gate. PM1 T cell cell lines were gated within the CD4^pos^ cells [60,61]. Data were analyzed by using FlowJo software (TreeStar).

### Blocking of HIV-1 binding to MDMs by anti-Siglec-7 masking antibody

MDMs were treated with 10 μg/ml of anti-Siglec-7 blocking polyclonal Ab or IgG isotype control (R&D System) in the presence of 20% human serum for 1 hour at 4°C. After three washing with PBS 2% FCS cells were infected as described below.

### HIV-1 infection of CD4+ cells

CD4+ PM1 T cells or primary CD4+ T cells were infected with either HIV-1_BaL_ or HIV-1_IIIB_ viruses. HIV-1 strains were propagated in phytohemagglutinin (PHA)-activated PBMC and collected from cell supernatant at peak times of HIV-RNA content. Cells (1 × 10^6^/ml, 2 × 10^5^/well) were inoculated with HIV-1 stock (5 × 10^5^ HIV-RNA copies/well) previously treated (for 1 hour at 37°C) with Siglec-7- or NKp44-Fc protein (10 μg/ml) and cultured at 37°C in 20% FCS RPMI 1640 medium. HIV-RNA was evaluated in cell supernatants after 3 days of culture.

### HIV-1 entry assay

After 4 hour incubation with HIV-virus (5 × 10^5^ copies/well) CD4+ T cells (1 × 10^6^/ml, 2 × 10^5^/well) or MDMs (5 × 10^5^ cells/ml, 5 × 10^5^ cells/well) were washed twice and treated with trypsin (2.5 mg/ml) for 10’ at 37°C to remove the extracellular virus adherent to the cell surface. After three additional washing steps with complete culture medium, cell lysates were prepared with NP40 Alternative 0.5% (Calbiochem-Merck) and the concentration of HIV-RNA was measured by real-time PCR.

### Real time PCR

The evaluation of HIV replication capacity was determined by measuring the HIV-RNA copy numbers in cell culture supernatants or, for entry assay, in the cell lysate after elimination of cell debris. In detail, viral HIV-1 RNA was extracted from supernatants or cell extracts using the automatic Easy Mag extractor (Biomerieux, Lyon, France) and quantified by real time PCR targeting a conserved region in the long terminal repeat (LTR) as previously reported [62].

### Statistical analysis

Paired t test was used to compare the HIV IIIB-beads binding to Siglec-7 Fc-chimera protein. One-way ANOVA statistical test followed by a Bonferroni post-test was used to compare HIV-RNA copies/ml within the different experimental settings, while Kruskal Wallis statistical test followed by a Dunn post-test to compare the serum levels of sSiglec-7 in the different groups of HIV infected patients. Pearson correlation test was used to correlate sSiglec-7 with HIV-RNA levels or CD4+ cell numbers.

## Abbreviations

Siglec-7: Sialic acid-binding immunoglobulin-like lectin 7; HIV-1: Human immunodeficiency virus type 1; gp120: Glycoprotein 120, AIDS, acquired immunodeficiency syndrome; CCR5: C-C chemokine receptor type 5; CXCR4: C-X-C chemokine receptor type 4; MDM: Monocyte derived macrophages; NK: Natural killer; ELISA: Enzyme-linked immune-sorbent assay; PCR: Polymerase chain reaction.

## Competing interests

The authors have no competing interest to declare.

## Authors’ contributions

SV, PL, MUM and DM designed the research and provided reagents. SV, DM, SP, KH JM and RC performed the experiments. SV collected and analyzed the data. AR and RM provided patients’ samples. MM provided reagents and helped with the experimental design. SV, MUM and DM wrote the manuscript. All authors read and approved the final manuscript.

## Supplementary Material

Additional file 1: Figure S1 Surface expression of CD64 (FCγRI) on PM1 cell line and CD4^pos^ primary T cells. Flow cytometric dot plot graphs showing the surface expression of CD64 (lower line) compared to the related isotypes (upper line) from a representative healthy donor out of three independent experiments performed. CD14^pos^ monocytes were chosen as positive control. Click here for file

Additional file 2: Figure S2 Surface expression of CD32 (FCγRII) on PM1 cell line and CD4^pos^ primary T cells. Flow cytometric dot plot graphs showing the surface expression of CD32 (lower line) compared to the related isotypes (upper line) from a representative healthy donor out of three independent experiments performed. CD14^pos^ monocytes were chosen as positive control. Click here for file

Additional file 3: Figure S3 Surface expression of CD1 6(FCγRIII) on PM1 cell line and CD4^pos^ primary T cells. Flow cytometric dot plot graphs showing the surface expression of CD16 (lower line) compared to the related isotypes (upper line) from a representative healthy donor out of three independent experiments performed. CD56^pos^ NK cells were chosen as positive control. Click here for file

## References

[B1] Pantophlet R Burton DR GP120: target for neutralizing HIV-1 antibodies Annu Rev Immunol 2006 10 739 769 10.1146/annurev.immunol.24.021605.090557 16551265

[B2] Scanlan CN Offer J Zitzmann N Dwek RA Exploiting the defensive sugars of HIV-1 for drug and vaccine design Nature 2007 10 1038 1045 10.1038/nature05818 17460665

[B3] Wei X Decker JM Wang S Hui H Kappes JC Wu X Salazar-Gonzalez JF Salazar MG Kilby JM Saag MS Antibody neutralization and escape by HIV-1 Nature 2003 10 307 312 10.1038/nature01470 12646921

[B4] Go EP Chang Q Liao HX Sutherland LL Alam SM Haynes BF Desaire H Glycosylation site-specific analysis of clade C HIV-1 envelope proteins J Proteome Res 2009 10 4231 4242 10.1021/pr9002728 19610667 PMC2756219

[B5] Zhang M Gaschen B Blay W Foley B Haigwood N Kuiken C Korber B Tracking global patterns of N-linked glycosylation site variation in highly variable viral glycoproteins: HIV, SIV, and HCV envelopes and influenza hemagglutinin Glycobiology 2004 10 1229 1246 10.1093/glycob/cwh106 15175256

[B6] Schauer R Achievements and challenges of sialic acid research Glycoconj J 2000 10 485 499 10.1023/A:1011062223612 11421344 PMC7087979

[B7] Varki A Glycan-based interactions involving vertebrate sialic-acid-recognizing proteins Nature 2007 10 1023 1029 10.1038/nature05816 17460663

[B8] Yamaji T Teranishi T Alphey MS Crocker PR Hashimoto Y A small region of the natural killer cell receptor, Siglec-7, is responsible for its preferred binding to alpha 2,8-disialyl and branched alpha 2,6-sialyl residues. A comparison with Siglec-9 J Biol Chem 2002 10 6324 6332 10.1074/jbc.M110146200 11741958

[B9] Nicoll G Ni J Liu D Klenerman P Munday J Dubock S Mattei MG Crocker PR Identification and characterization of a novel siglec, siglec-7, expressed by human natural killer cells and monocytes J Biol Chem 1999 10 34089 34095 10.1074/jbc.274.48.34089 10567377

[B10] Crocker PR Paulson JC Varki A Siglecs and their roles in the immune system Nat Rev Immunol 2007 10 255 266 10.1038/nri2056 17380156

[B11] von Gunten S Bochner BS Basic and clinical immunology of Siglecs Ann N Y Acad Sci 2008 10 61 82 10.1196/annals.1443.011 19076345 PMC3902170

[B12] Avril T Floyd H Lopez F Vivier E Crocker PR The membrane-proximal immunoreceptor tyrosine-based inhibitory motif is critical for the inhibitory signaling mediated by Siglecs-7 and -9, CD33-related Siglecs expressed on human monocytes and NK cells J Immunol 2004 10 6841 6849 15557178 10.4049/jimmunol.173.11.6841

[B13] Varchetta S Brunetta E Roberto A Mikulak J Hudspeth KL Mondelli MU Mavilio D Engagement of Siglec-7 receptor induces a pro-inflammatory response selectively in monocytes PLoS One 2012 10 e45821 10.1371/journal.pone.0045821 23029261 PMC3461047

[B14] Avril T Wagner ER Willison HJ Crocker PR Sialic acid-binding immunoglobulin-like lectin 7 mediates selective recognition of sialylated glycans expressed on Campylobacter jejuni lipooligosaccharides Infect Immun 2006 10 4133 4141 10.1128/IAI.02094-05 16790787 PMC1489752

[B15] Khatua B Ghoshal A Bhattacharya K Mandal C Saha B Crocker PR Mandal C Sialic acids acquired by Pseudomonas aeruginosa are involved in reduced complement deposition and siglec mediated host-cell recognition FEBS Lett 2010 10 555 561 10.1016/j.febslet.2009.11.087 19945458 PMC3640159

[B16] Khatua B Bhattacharya K Mandal C Sialoglycoproteins adsorbed by Pseudomonas aeruginosa facilitate their survival by impeding neutrophil extracellular trap through siglec-9 J Leukoc Biol 2012 10 641 655 10.1189/jlb.0511260 22241833

[B17] Mercier S St-Pierre C Pelletier I Ouellet M Tremblay MJ Sato S Galectin-1 promotes HIV-1 infectivity in macrophages through stabilization of viral adsorption Virology 2008 10 121 129 10.1016/j.virol.2007.09.034 18028978

[B18] Ouellet M Mercier S Pelletier I Bounou S Roy J Hirabayashi J Sato S Tremblay MJ Galectin-1 acts as a soluble host factor that promotes HIV-1 infectivity through stabilization of virus attachment to host cells J Immunol 2005 10 4120 4126 15778371 10.4049/jimmunol.174.7.4120

[B19] Furci L Sironi F Tolazzi M Vassena L Lusso P Alpha-defensins block the early steps of HIV-1 infection: interference with the binding of gp120 to CD4 Blood 2007 10 2928 2935 17132727 10.1182/blood-2006-05-024489

[B20] Feng Z Dubyak GR Lederman MM Weinberg A Cutting edge: human beta defensin 3–a novel antagonist of the HIV-1 coreceptor CXCR4 J Immunol 2006 10 782 786 16818731 10.4049/jimmunol.177.2.782

[B21] Munk C Wei G Yang OO Waring AJ Wang W Hong T Lehrer RI Landau NR Cole AM The theta-defensin, retrocyclin, inhibits HIV-1 entry AIDS Res Hum Retroviruses 2003 10 875 881 10.1089/088922203322493049 14585219

[B22] Ezekowitz RA Kuhlman M Groopman JE Byrn RA A human serum mannose-binding protein inhibits in vitro infection by the human immunodeficiency virus J Exp Med 1989 10 185 196 10.1084/jem.169.1.185 2909656 PMC2189190

[B23] de Witte L Nabatov A Pion M Fluitsma D de Jong MA de Gruijl T Piguet V van Kooyk Y Geijtenbeek TB Langerin is a natural barrier to HIV-1 transmission by Langerhans cells Nat Med 2007 10 367 371 10.1038/nm1541 17334373

[B24] Geijtenbeek TB Kwon DS Torensma R van Vliet SJ van Duijnhoven GC Middel J Cornelissen IL Nottet HS KewalRamani VN Littman DR DC-SIGN, a dendritic cell-specific HIV-1-binding protein that enhances trans-infection of T cells Cell 2000 10 587 597 10.1016/S0092-8674(00)80694-7 10721995

[B25] Nguyen DG Hildreth JE Involvement of macrophage mannose receptor in the binding and transmission of HIV by macrophages Eur J Immunol 2003 10 483 493 10.1002/immu.200310024 12645947

[B26] de Witte L Bobardt M Chatterji U Degeest G David G Geijtenbeek TB Gallay P Syndecan-3 is a dendritic cell-specific attachment receptor for HIV-1 Proc Natl Acad Sci USA 2007 10 19464 19469 10.1073/pnas.0703747104 18040049 PMC2148312

[B27] Lambert AA Gilbert C Richard M Beaulieu AD Tremblay MJ The C-type lectin surface receptor DCIR acts as a new attachment factor for HIV-1 in dendritic cells and contributes to trans- and cis-infection pathways Blood 2008 10 1299 1307 10.1182/blood-2008-01-136473 18541725 PMC2515113

[B28] Rempel H Calosing C Sun B Pulliam L Sialoadhesin expressed on IFN-induced monocytes binds HIV-1 and enhances infectivity PLoS One 2008 10 e1967 10.1371/journal.pone.0001967 18414664 PMC2288672

[B29] Zou Z Chastain A Moir S Ford J Trandem K Martinelli E Cicala C Crocker P Arthos J Sun PD Siglecs facilitate HIV-1 infection of macrophages through adhesion with viral sialic acids PLoS One 2011 10 e24559 10.1371/journal.pone.0024559 21931755 PMC3169630

[B30] Izquierdo-Useros N Lorizate M Puertas MC Rodriguez-Plata MT Zangger N Erikson E Pino M Erkizia I Glass B Clotet B Siglec-1 is a novel dendritic cell receptor that mediates HIV-1 trans-infection through recognition of viral membrane gangliosides PLoS Biol 2012 10 e1001448 10.1371/journal.pbio.1001448 23271952 PMC3525531

[B31] Brunetta E Fogli M Varchetta S Bozzo L Hudspeth KL Marcenaro E Moretta A Mavilio D The decreased expression of Siglec-7 represents an early marker of dysfunctional natural killer-cell subsets associated with high levels of HIV-1 viremia Blood 2009 10 3822 3830 10.1182/blood-2009-06-226332 19710502 PMC2773483

[B32] Lusso P Cocchi F Balotta C Markham PD Louie A Farci P Pal R Gallo RC Reitz MS Jr Growth of macrophage-tropic and primary human immunodeficiency virus type 1 (HIV-1) isolates in a unique CD4+ T-cell clone (PM1): failure to downregulate CD4 and to interfere with cell-line-tropic HIV-1 J Virol 1995 10 3712 3720 7745720 10.1128/jvi.69.6.3712-3720.1995 PMC189087

[B33] Hudspeth K Fogli M Correia DV Mikulak J Roberto A Della Bella S Silva-Santos B Mavilio D Engagement of NKp30 on Vdelta1 T cells induces the production of CCL3, CCL4, and CCL5 and suppresses HIV-1 replication Blood 2012 10 4013 4016 10.1182/blood-2011-11-390153 22403253

[B34] Dalgleish AG Beverley PC Clapham PR Crawford DH Greaves MF Weiss RA The CD4 (T4) antigen is an essential component of the receptor for the AIDS retrovirus Nature 1984 10 763 767 10.1038/312763a0 6096719

[B35] Klatzmann D Champagne E Chamaret S Gruest J Guetard D Hercend T Gluckman JC Montagnier L T-lymphocyte T4 molecule behaves as the receptor for human retrovirus LAV Nature 1984 10 767 768 10.1038/312767a0 6083454

[B36] McLain L Dimmock NJ A human CD4+ T-cell line expresses functional CD64 (Fc gamma RI), CD32 (Fc gamma RII), and CD16 (Fc gamma RIII) receptors but these do not enhance the infectivity of HIV-1-IgG complexes Immunology 1997 10 109 114 10.1046/j.1365-2567.1997.00116.x 9038720 PMC1456708

[B37] Lanier LL Kipps TJ Phillips JH Functional properties of a unique subset of cytotoxic CD3+ T lymphocytes that express Fc receptors for IgG (CD16/Leu-11 antigen) J Exp Med 1985 10 2089 2106 10.1084/jem.162.6.2089 2415663 PMC2187997

[B38] Kazazi F Mathijs JM Foley P Cunningham AL Variations in CD4 expression by human monocytes and macrophages and their relationships to infection with the human immunodeficiency virus J Gen Virol 1989 10 Pt 10 2661 2672 2677236 10.1099/0022-1317-70-10-2661

[B39] Wilen CB Tilton JC Doms RW HIV: cell binding and entry Cold Spring Harb Perspect Med 2012 10 8 doi:pii: a006866. 10.1101/cshperspect.a006866. Review 10.1101/cshperspect.a006866 PMC3405824 22908191

[B40] Jacobs T Erdmann H Fleischer B Molecular interaction of Siglecs (sialic acid-binding Ig-like lectins) with sialylated ligands on Trypanosoma cruzi Eur J Cell Biol 2010 10 113 116 10.1016/j.ejcb.2009.10.006 19910077

[B41] Erdmann H Steeg C Koch-Nolte F Fleischer B Jacobs T Sialylated ligands on pathogenic Trypanosoma cruzi interact with Siglec-E (sialic acid-binding Ig-like lectin-E) Cell Microbiol 2009 10 1600 1611 10.1111/j.1462-5822.2009.01350.x 19552697

[B42] Carlin AF Lewis AL Varki A Nizet V Group B streptococcal capsular sialic acids interact with siglecs (immunoglobulin-like lectins) on human leukocytes J Bacteriol 2007 10 1231 1237 10.1128/JB.01155-06 16997964 PMC1797352

[B43] Carlin AF Uchiyama S Chang YC Lewis AL Nizet V Varki A Molecular mimicry of host sialylated glycans allows a bacterial pathogen to engage neutrophil Siglec-9 and dampen the innate immune response Blood 2009 10 3333 3336 10.1182/blood-2008-11-187302 19196661 PMC2665898

[B44] Jones C Virji M Crocker PR Recognition of sialylated meningococcal lipopolysaccharide by siglecs expressed on myeloid cells leads to enhanced bacterial uptake Mol Microbiol 2003 10 1213 1225 10.1046/j.1365-2958.2003.03634.x 12940982

[B45] Pillai S Netravali IA Cariappa A Mattoo H Siglecs and immune regulation Annu Rev Immunol 2012 10 357 392 10.1146/annurev-immunol-020711-075018 22224769 PMC3781015

[B46] Cao H Crocker PR Evolution of CD33-related siglecs: regulating host immune functions and escaping pathogen exploitation? Immunology 2011 10 18 26 10.1111/j.1365-2567.2010.03368.x 21070233 PMC3015071

[B47] Vanderheijden N Delputte PL Favoreel HW Vandekerckhove J Van Damme J van Woensel PA Nauwynck HJ Involvement of sialoadhesin in entry of porcine reproductive and respiratory syndrome virus into porcine alveolar macrophages J Virol 2003 10 8207 8215 10.1128/JVI.77.15.8207-8215.2003 12857889 PMC165228

[B48] Delputte PL Nauwynck HJ Porcine arterivirus infection of alveolar macrophages is mediated by sialic acid on the virus J Virol 2004 10 8094 8101 10.1128/JVI.78.15.8094-8101.2004 15254181 PMC446125

[B49] Sun J Barbeau B Sato S Tremblay MJ Neuraminidase from a bacterial source enhances both HIV-1-mediated syncytium formation and the virus binding/entry process Virology 2001 10 26 36 10.1006/viro.2001.0889 11352665

[B50] Kwong PD Wyatt R Sattentau QJ Sodroski J Hendrickson WA Oligomeric modeling and electrostatic analysis of the gp120 envelope glycoprotein of human immunodeficiency virus J Virol 2000 10 1961 1972 10.1128/JVI.74.4.1961-1972.2000 10644369 PMC111674

[B51] Sakaida H Hori T Yonezawa A Sato A Isaka Y Yoshie O Hattori T Uchiyama T T-tropic human immunodeficiency virus type 1 (HIV-1)-derived V3 loop peptides directly bind to CXCR-4 and inhibit T-tropic HIV-1 infection J Virol 1998 10 9763 9770 9811711 10.1128/jvi.72.12.9763-9770.1998 PMC110487

[B52] Crocker PR Varki A Siglecs in the immune system Immunology 2001 10 137 145 10.1046/j.0019-2805.2001.01241.x 11412300 PMC1783234

[B53] Brunetta E Fogli M Varchetta S Bozzo L Hudspeth KL Marcenaro E Moretta A Mavilio D Chronic HIV-1 viremia reverses NKG2A/NKG2C ratio on natural killer cells in patients with human cytomegalovirus co-infection AIDS 2010 10 27 34 10.1097/QAD.0b013e3283328d1f 19910789

[B54] Brunetta E Hudspeth KL Mavilio D Pathologic natural killer cell subset redistribution in HIV-1 infection: new insights in pathophysiology and clinical outcomes J Leukoc Biol 2010 10 1119 1130 10.1189/jlb.0410225 20651298

[B55] Lackner AA Lederman MM Rodriguez B HIV pathogenesis: the host Cold Spring Harb Perspect Med 2012 10 a007005 22951442 10.1101/cshperspect.a007005 PMC3426821

[B56] Shacklett BL Immune responses to HIV and SIV in mucosal tissues: 'location, location, location' Curr Opin HIV AIDS 2010 10 128 134 10.1097/COH.0b013e328335c178 20543589 PMC2886278

[B57] Brenchley JM Price DA Schacker TW Asher TE Silvestri G Rao S Kazzaz Z Bornstein E Lambotte O Altmann D Microbial translocation is a cause of systemic immune activation in chronic HIV infection Nat Med 2006 10 1365 1371 17115046 10.1038/nm1511

[B58] Hudspeth K Silva-Santos B Mavilio D Natural cytotoxicity receptors: broader expression patterns and functions in innate and adaptive immune cells Front Immunol 2013 10 69 23518691 10.3389/fimmu.2013.00069 PMC3603285

[B59] Eisele E Siliciano RF Redefining the viral reservoirs that prevent HIV-1 eradication Immunity 2012 10 377 388 10.1016/j.immuni.2012.08.010 22999944 PMC3963158

[B60] Gupta N Arthos J Khazanie P Steenbeke TD Censoplano NM Chung EA Cruz CC Chaikin MA Daucher M Kottilil S Targeted lysis of HIV-infected cells by natural killer cells armed and triggered by a recombinant immunoglobulin fusion protein: implications for immunotherapy Virology 2005 10 491 497 10.1016/j.virol.2004.12.018 15680414

[B61] Rusmini M Griseri P Lantieri F Matera I Hudspeth KL Roberto A Mikulak J Avanzini S Rossi V Mattioli G Induction of RET dependent and independent pro-inflammatory programs in human peripheral blood mononuclear cells from Hirschsprung patients PLoS One 2013 10 e59066 10.1371/journal.pone.0059066 23527089 PMC3601093

[B62] Avettand-Fenoel V Chaix ML Blanche S Burgard M Floch C Toure K Allemon MC Warszawski J Rouzioux C LTR real-time PCR for HIV-1 DNA quantitation in blood cells for early diagnosis in infants born to seropositive mothers treated in HAART area (ANRS CO 01) J Med Virol 2009 10 217 223 10.1002/jmv.21390 19107966

