# Supplementary figures and images for: Sialic acid-binding Ig-like lectin-7 interacts with HIV-1 gp120 and facilitates infection of CD4pos T cells and macrophages
Source: Retrovirology. 2013 Dec 13;10:154. doi: 10.1186/1742-4690-10-154 (PMC3878752; doi:10.1186/1742-4690-10-154)

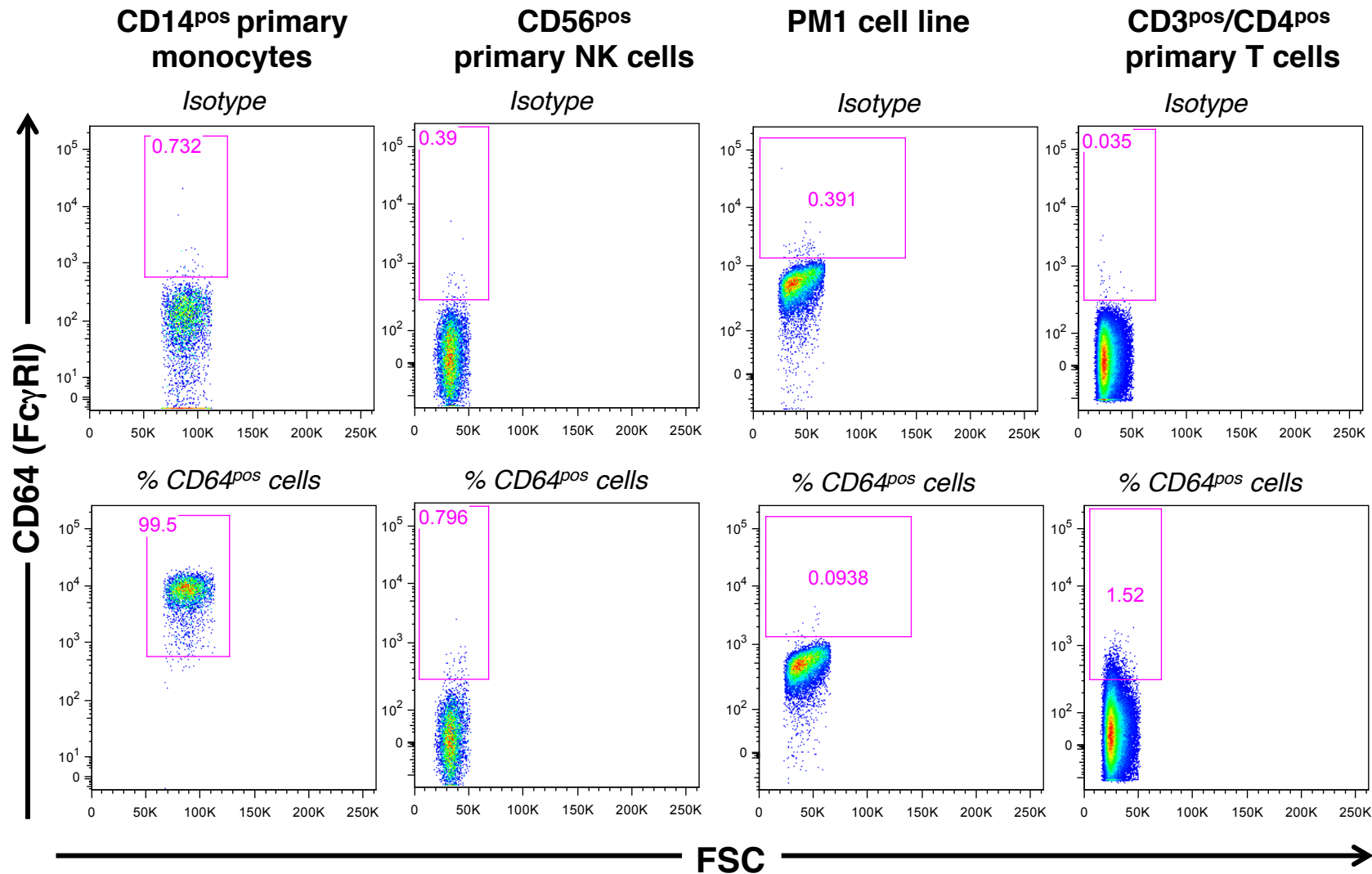

Varchetta S. et al.  
Supplemental Figure 1  
(Additional File 1)

Supplement: Additional file 1: Figure S1 — Surface expression of CD64 (FCγRI) on PM1 cell line and CD4pos primary T cells. Flow cytometric dot plot graphs showing the surface expression of CD64 (lower line) compared to the related isotypes (upper line) from a representative healthy donor out of three independent experiments performed. CD14pos monocytes were chosen as positive control. [file 1742-4690-10-154-S1.pdf]

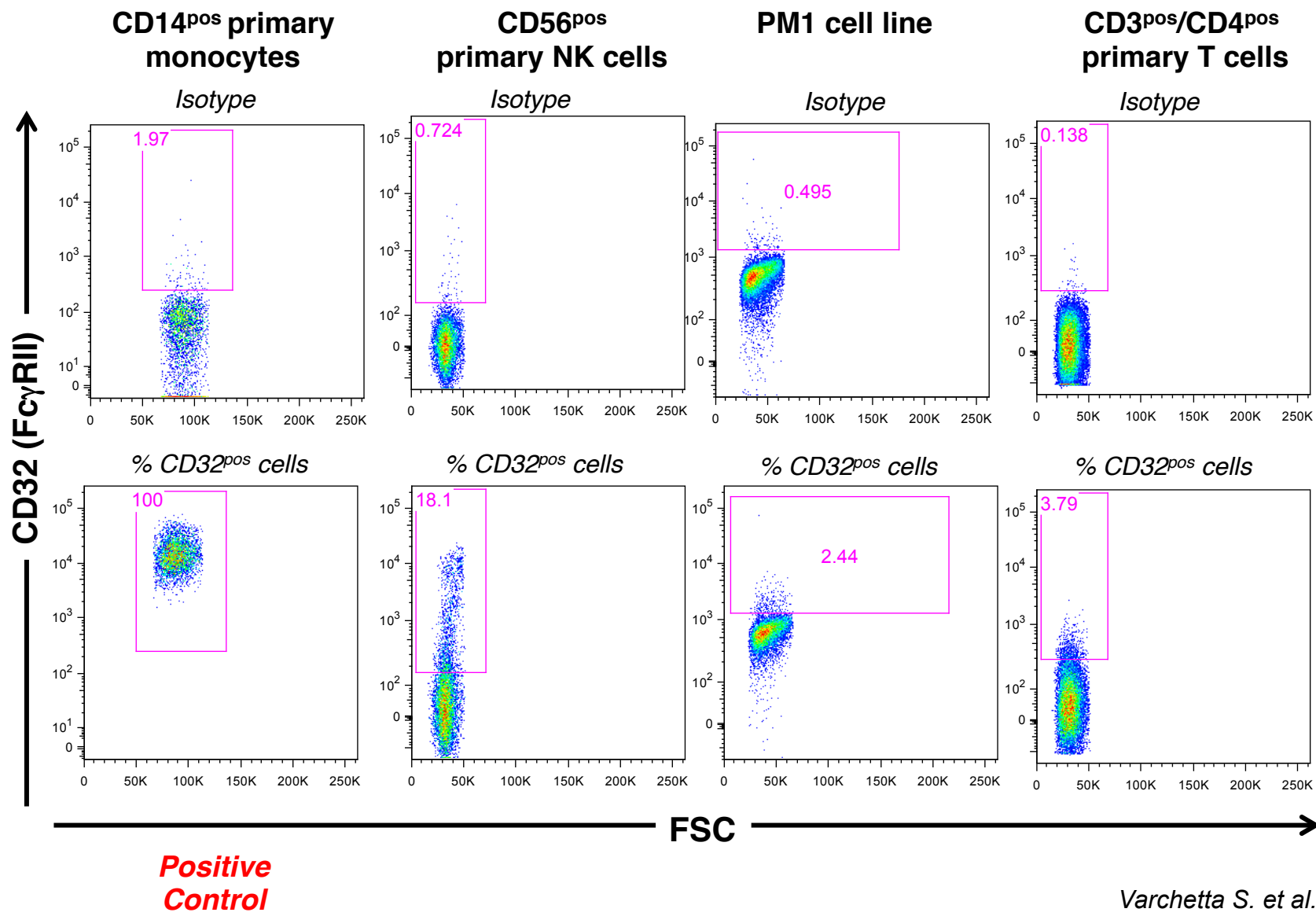

Varchetta S. et al.  
Supplemental Figure 2  
(Additional File 2)

Supplement: Additional file 2: Figure S2 — Surface expression of CD32 (FCγRII) on PM1 cell line and CD4pos primary T cells. Flow cytometric dot plot graphs showing the surface expression of CD32 (lower line) compared to the related isotypes (upper line) from a representative healthy donor out of three independent experiments performed. CD14pos monocytes were chosen as positive control. [file 1742-4690-10-154-S2.pdf]

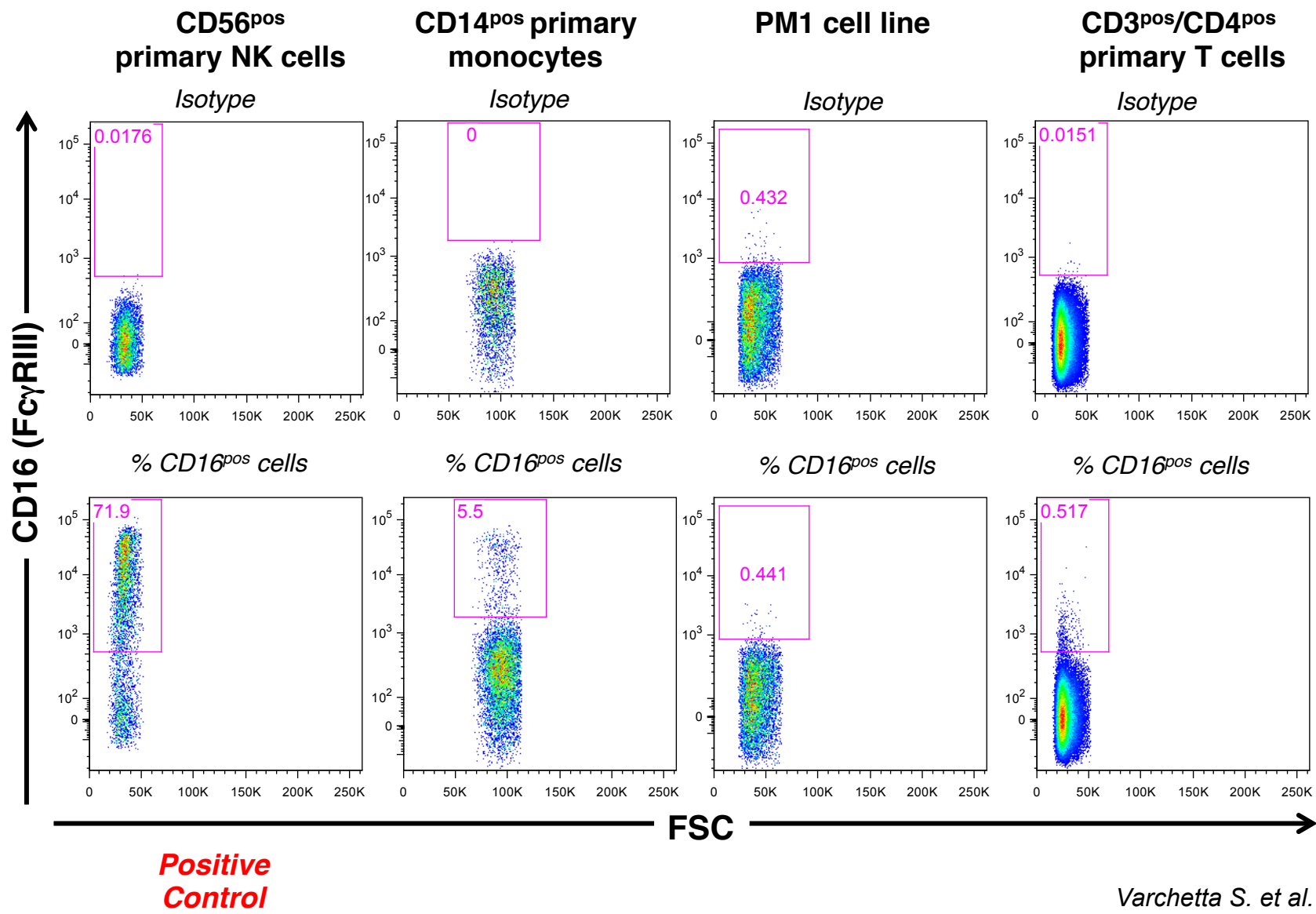

Varchetta S. et al.  
Supplemental Figure 3  
(Additional File 3)

Supplement: Additional file 3: Figure S3 — Surface expression of CD1 6(FCγRIII) on PM1 cell line and CD4pos primary T cells. Flow cytometric dot plot graphs showing the surface expression of CD16 (lower line) compared to the related isotypes (upper line) from a representative healthy donor out of three independent experiments performed. CD56pos NK cells were chosen as positive control. [file 1742-4690-10-154-S3.pdf]
